# Developing a “Fast-Track” Strategy for Interventional Management of Patients With Idiopathic Intracranial Hypertension

**DOI:** 10.3389/fopht.2022.923092

**Published:** 2022-06-20

**Authors:** Shahnaz Miri, Abhay Moghekar, Andrew R. Carey, Phillipe Gailloud, Neil R. Miller

**Affiliations:** ^1^ Department of Ophthalmology, Wilmer Eye Institute, Johns Hopkins University School of Medicine, Baltimore, MD, United States; ^2^ Departments of Neurology and Neurosurgery, Johns Hopkins University School of Medicine, Baltimore, MD, United States; ^3^ Division of Interventional Neuroradiology and Department of Art as Applied to Medicine, Johns Hopkins University School of Medicine, Baltimore, MD, United States

**Keywords:** idiopathic intracranial hypertension (IIH), CSF shunting, cerebral venous sinus stenting, fast-track protocol, surgical intervention

## Abstract

Idiopathic intracranial hypertension (IIH) has an increasing incidence worldwide over the past decade, with a high economic burden on patients and society. Up to 10% of patients with IIH have progressive visual decline requiring an invasive intervention (including cerebrospinal fluid shunting, cerebral dural sinus stenting, or optic nerve sheath fenestration [ONSF]). IIH patients with visual decline usually undergo evaluation and initial management through the emergency department (ED) and commonly have a long hospital stay due to the lack of a dedicated methodology for evaluation and management, particularly in patients who present with visual loss (i.e., fulminant IIH). An innovative practice approach is needed to improve the means of multidisciplinary communication in care and evaluation of IIH patients. This paper aims to discuss the need for the development and implementation of a multidisciplinary “fast-track” strategy for the evaluation and management of patients with fulminant IIH or those with a suboptimal response to maximum tolerated medical treatment at risk for visual loss. We suggest that such a program could reduce hospital stay and ED visits and therefore reduce healthcare costs and improve patient outcomes by accelerating the management process.

## Introduction

Idiopathic intracranial hypertension (IIH) is characterized by elevated intracranial pressure (ICP) with normal cerebrospinal fluid (CSF) content without underlying etiology. It is often associated with headache, papilledema, and visual decline. IIH has a reported incidence of 2.4/100,000 (2002–2014) in the US, 3.3/100,000 in women, and 22.0/100,000 among obese women aged 15 to 44 years (1). The incidence of IIH has reportedly risen from 2.3 to 4.7 per 100,000 between 2002 and 2016 in the UK (2). Risk factors for IIH are well-known and include obesity or weight gain, female gender, and endocrine disorders (3).

Fulminant IIH, defined as a precipitous decline in visual function within 4 weeks of diagnosis, has been reported in less than 10% of patients who require invasive interventions (2) including CSF shunting (6.4%), cerebral dural sinus stenting (1%), and optic nerve sheath fenestration (0.5%) (4). Patients with primary shunt placement had a 23.7% rate of 30 days emergency readmission. Patients with neurovascular stent and ONSF had 10.0% and 9.74% rate of 30 days readmission, retrospectively (4).

With the increasing incidence of IIH, there is an increasing burden of healthcare costs for patients and society. The admission and readmission rates of patients with IIH have increased significantly over the past decade (442% increase between 2002 and 2014) with an average of 2.7 days duration of hospital stay without surgical intervention and 8.8 days for those who undergo a shunt procedure (2). The reported hospital admission rate for IIH patients was 38% in 2007 (5). In 2014, the inpatient cost of IIH management in the UK was reported to be £50 million (about US$55 million) per annum and is estimated to be £462 million (about US$510 million) per annum by 2030 (2). The direct and indirect costs of hospital admission for IIH patients are four times greater than population-based per-person admissions (considering the significant loss of productivity in these young working-age patients), with a total economic cost of $444 million in the US in 2007 (5).

Establishing a fast-track evaluation of patients with fulminant IIH, with a multidisciplinary approach in management and rapid arrangement of surgical intervention for these patients, can reduce the healthcare cost and burden and potentially improve clinical outcome. The concept of “Fast-track” strategy in the evaluation of surgical patients was first initiated in the 1990s and since then there have been numerous studies supporting fast-track strategies over conventional methods in the evaluation and management of such patients (6). A multidisciplinary team approach defined in fast-track strategy protocols has proven to reduce the length of hospital stay and risk of complications. There has been continuous evolution of a fast-track strategy in other fields with improvement of interdisciplinary collaboration, pre-admission counseling, preoperative evaluation and preparation, standard medical protocols, postoperative counseling and nutritional care, early mobilization, and discharge planning.

## IIH Initial Evaluation and Surgical Management Indications

There is a lack of high-quality evidence for the investigation and management of IIH, with current guidelines being based on consensus rather than on prospective, controlled clinical trials (7). The recommended evaluation of patients with papilledema includes assessing pupillary responses, measuring best-corrected visual acuity, formal visual field testing, dilated funduscopic exam and optical coherence tomography to determine severity of papilledema, and exclusion of other causes of optic disc elevation [such as buried optic disc drusen), followed by neuroimaging [brain computed tomography (CT) and CT venography (CTV) or magnetic resonance imaging [MRI] and MR venography (MRV)]shortly after the diagnosis of papilledema. If no intracranial lesion is identified, lumbar puncture (LP) and CSF analysis are the next step in the evaluation. The diagnosis is established in patients when the opening pressure is ≥25 cm of H_2_O (≥28 cm in children) and the CSF has normal protein and glucose concentrations and no cells. The management of IIH should address the underlying modifiable risk factors (e.g., obesity and sleep apnea), monitor visual function by regular assessment, treat elevated ICP, and address headache comorbidity (7). Cases with imminent visual risk (patients with high-grade disc edema and severe or rapidly progressive vision loss) are categorized as fulminant IIH (7).

Surgical interventions indicated in IIH cases with imminent vision risk require prompt aggressive management for acute reduction in ICP to preserve vision. Interventions include CSF diversion with shunts, cerebral dural sinus stenting, and/or ONSF. If immediate surgery (within 24 h of diagnosis of fulminant IIH) is not feasible, a temporizing lumbar drain should be considered in an inpatient setting. Daily LPs with close visual function monitoring may serve as an alternative (8). Another indication for urgent surgical intervention is lack of response to maximally tolerated or maximum medical therapy and/or deterioration of visual function at follow-up examinations (7).

Ventriculoperitoneal shunt (VPS), ventriculoatrial shunt (VAS), and lumboperitoneal shunt (LPS) are the most widely used surgical methods for ICP reduction in IIH patients. They act by diverting CSF from one of the lateral ventricles to the peritoneal or atrial (cardiac) space. CSF shunting improves headache in 60% to 90% of patients and improves visual functions in 40% to 100% (9).

Cerebral dural sinus stenting is a relatively new treatment option that can be considered when significant bilateral or dominant unilateral dural venous stenosis (usually in the transverse sinus) is identified in patients with vision-threatening IIH or those with suboptimal response to medical treatment. In our opinion, CTV is the most sensitive imaging modality to establish the presence and degree of stenosis, but MRV also has excellent sensitivity. Thus, the decision as to what method to use should depend on the amount of experience the imaging center has with each technique. The functional significance of the stenosis then can be confirmed by manometry. In cases with a high pressure gradient across the region of stenosis, dural venous sinus stenting is the treatment of choice as it has low rates of complications and revisions, a satisfactory clinical outcome with respect to headache, tinnitus, papilledema and visual outcome, and long-term stent patency (10–12). For example, based on a systematic review and meta-analysis of 20 articles from 18 different centers, including 474 patients with dural sinus stenting for IIH, the rate of major complications was 1.9% (13). In another meta-analysis that included 136 patients with IIH who underwent dural sinus stenting, rates of improvement in papilledema, visual function, and headache were 97%, 78%, and 83%, respectively (10).

There is no initial cost difference between CSF shunting and venous stent procedures. However, dural venous sinus stenting costs may be significantly less in the long term, given the high rate of shunt failure and the frequent need for revision (14).

ONSF is a surgical method used by ophthalmic surgeons that rapidly can improve visual acuity and visual fields in patients with high-grade papilledema and visual decline (15). However, it does not effectively treat headache, with a rate of improvement in one meta-analysis being only 44% compared with 80% improvement with CSF diversion procedures and 83% with venous sinus stenting (10).

## Emergency Visits and Hospital Readmission in Patients With Known IIH

A single-center study on emergency department (ED) utilization among patients with a known diagnosis of IIH showed that 39% of IIH patients used emergency services over the study period 2010–2012 (16). In another large population-based study in the UK, 37.2% of patients admitted for IIH between 2002 to 2019 had one or more subsequent hospital admissions in the following year (4). The mean 30-day emergency readmission rate following CSF shunting was reported to be 23.1% (4).

Another study of ED visits of IIH patients with a history of CSF shunting showed that shunt series x-rays detected catheter pathology in 3.9% of ED visits. This study suggests that x-rays may not be a useful screening tool in detecting shunt malfunction in IIH patients in the ED (17). A break in shunt catheter that can be detected on x-rays is a rare reason for shunt malfunction in IIH.

Consensus on close monitoring and patient follow-up after diagnosis of IIH is important to improve patient outcomes, reduce ED visits and hospital admissions, and reduce healthcare costs. Creating a multidisciplinary team approach including neurologists, ophthalmologists or neuro-ophthalmologists, neurosurgeons, and diagnostic and interventional neuroradiologists is the key to achieving this goal.

## Fast-Track IIH Care for Patient With Fulminant or Medically Refractory Disease

“Fast-track” intervention refers to an interdisciplinary management strategy that focuses on preoperative patient education, optimization of operative field and anesthesia methods, and enforced postoperative convalescence (18). A “fast-track” methodology in other surgical specialties has provided a major enhancement in recovery, decreasing hospital stay, and reduction of general morbidity in a variety of procedures (19). For example, studies on elective fast-track endovascular aneurysm repair (EVAR), defined by using a 14 Fr stent graft, bilateral percutaneous access, no general anesthesia or intensive care monitoring, and next-day hospital discharge, show superiority and improvement of care utilization, faster discharge, and lower readmission rate, over standard EVAR methods (20). “Fast-track” protocols have also been developed and implemented successfully in patients with neurosurgical conditions including subarachnoid hemorrhage and pituitary tumor surgery, leading to shorter hospital stays, reduced healthcare costs, and improvement of outpatient surveillance and monitoring (21, 22).

To date, there has been no study on a fast-track strategy for IIH patients who require invasive management. Implementing a multidisciplinary strategy for accelerated visual function assessment, neuroimaging, LP, and early surgical consultation and planning in cases of fulminant IIH or medically refractory disease are critical steps in such a strategy. Hospitals should establish a team of “fast-track IIH care” for rapid assessment of IIH patients presenting to the ED or in neurology or ophthalmology outpatient clinics. This interdisciplinary team would facilitate direct admission or accelerated elective admissions for cases in need of urgent interventions while preventing unnecessary ED visits in cases with no visual compromise.

Periprocedural patient education and close post-operative outpatient monitoring can improve recovery and clinical outcomes. Patient education on potential post-discharge problems may prevent readmission and ED visit rate. It remains critical to address the underlying risk factors that contributed to IIH as all invasive interventions can potentially fail. CSF shunts can become obstructed, break, or develop infections, ONSF may lose efficacy over time, and stents can develop thromboses or pre-stent or post-stent stenoses. Nutritional consultation for obese patients and bariatric surgical consultation for those with morbid obesity can be included in the fast-track IIH care. Sleep apnea screening and treatment can be another component of this strategy.

## Proposed Components of a Fast-Track IIH Care

Formulating a standard fast-track IIH care model in the hospital or outpatient setting can benefit patients with IIH, regardless of their location or socioeconomic status. The first step in defining the model is to establish a team that includes a committed care coordinator who has a direct line of communication for receiving referrals, triaging patients, and contacting the specific members of the team who would be involved in the urgent/emergent management of the patient. The position of care coordinator could be a physician, registered nurse, nurse practitioner, or physician assistant. The rest of the team would include at least one neurologist, neuro-ophthalmologist, neurosurgeon, and interventional radiologist, all of whom are committed to being available on an urgent or emergent basis to assess and manage the patient.

We propose that a fast-track protocol should address the following components (with the caveat that it will need to be validated prospectively in trials).

1. Establish risk of vision loss by papilledema grading, OCT if available, and formal quantitative assessment of afferent visual function with some details about what centers lacking access to perimetry or neuro-ophthalmologists can do.
2. Establish a dedicated “IIH protocol”—MRI-MRV or CT-CTV to establish the lack of structural pathology and document cerebral Dural sinus stenosis
3. LP to measure opening pressure accurately and to exclude secondary causes of raised ICP.
4. Decision tree regarding optimum treatment should be implemented (will depend on the personnel at each center). If no intervention can be performed within an appropriate time period, admitting the patient to the hospital for a temporary lumbar drain or daily LPs with close monitoring of visual function is preferable to discharge from an ED and outpatient follow-up.
5. If experienced neuro-ophthalmologists or neurosurgeons are accessible, ONSF or CSF shunting are options. If MRV/CTV shows severe bilateral or dominant unilateral cerebral venous sinus stenosis, and an experienced interventional neuroradiologist is available, cerebral venous sinus stenting is an option
6. A post-discharge plan to address underlying risk factors and continue to monitor visual function closely.

Future studies are necessary to investigate the feasibility and influence of an interdisciplinary fast-track strategy on clinical recovery, particularly visual outcomes, the length of hospital stay, overall costs of care, and the rate of readmissions in patients with IIH.

## Advantages for Physicians of a Fast-Track IIH Care Strategy

A fast-track care strategy for patients with IIH can be particularly helpful for both the patients and their physicians, whether in the community or even in a tertiary care setting. For example, it can be difficult for an optometrist, neurologist, or ophthalmologist in a community setting to determine the optimum intervention in a patient with IIH and the urgency with which that intervention is needed. Although true fulminant IIH is not common, patients with what seems like “mild” IIH nevertheless can lose vision rapidly because of delays in their evaluation and management. Even if the physician is a faculty member at a tertiary care facility, it may be difficult for her/him to know exactly whom to contact, to whom to refer the patient, and how soon the patient needs to be evaluated. A well-publicized fast-track program consisting of a committed interdisciplinary team thus can be the key in determining the best treatment strategy for each individual patient. Some hospitals do not have direct access to such experts, and referral to outside facilities can be a cumbersome process. However, once a fast-track process is established at a particular facility, both community physicians and physicians at other locations can be notified of the availability of the program and counseled as to how to refer their patients for urgent/emergent care.

## Challenges in Implementing a Fast-Track IHH Care Strategy

From an organizational scope, implementing a fast-track IIH care strategy may encounter hesitation due to restrictions imposed by the new protocol, fear of alteration and adherence to the new setting as opposed to the traditional practice, difficulty with interdisciplinary cooperation, and reluctance in implementing externally guided protocols. Other challenges in implementation of fast-track approaches include logistic difficulties, time-consuming expenditure, as well as limitations in the healthcare system (23). Experts in different specialties in each institution must be actively involved in setting up such protocols to ensure their agreement with and commitment to the process.

A delay in neuroimaging and long wait times in the ED for initial neuroimaging are other barriers that may lead to delays in treatment as well as longer hospital stays and poor visual outcomes. Improvements in organizational imaging protocols, such as arranging accelerated CT/CTV and MRI/MRV protocols as soon as a patient with papilledema has been identified in the ED or in an outpatient clinic, can also facilitate early and appropriate evaluation and management of IIH patients, particularly those requiring urgent treatment. For this challenge, the presence of an interventional neuroradiologist or neurosurgeon to the interdisciplinary fast-track IIH care team can significantly accelerate IIH evaluation and management and may reduce hospital admissions and ED visits.

Patient-dependent factors may also complicate the implementation of fast-track strategies. Patient education and inclusion should be integrated into the fast-track concept to improve compliance and address underlying risk factors.

## Conclusion

The management of fulminant IIH or medically refractory patients with progressive visual function decline requires an interdisciplinary approach for selecting the appropriate treatment option and timing for intervention. Defining a fast-track IIH care concept with involvement of a multidisciplinary protocol can potentially improve care, reduce length of hospital stays and ED visits, improve patient outcomes, and reduce the economic burden of this growing population health concern. The successful implementation of the proposed fast-track IIH care requires interdisciplinary team collaboration, continuous training, and a positive feedback culture, which subsequently improves adherence to the protocol. To achieve wider implementation, a prospective study comparing this fast-track protocol with standard of care will need to be undertaken.

## Data Availability Statement

The original contributions presented in the study are included in the article. Further inquiries can be directed to the corresponding author.

## Author Contributions

SM contributed to original drafting of the manuscript and conceptualization. NRM, AM, ARC, and PG contributed to conceptualization, review, and editing the manuscript. All authors reviewed and approved the final submitted version.

## Conflict of Interest

SM has received funding for salary support from The Knights Templar Eye Foundation Inc. Professor of Ophthalmology.

The remaining authors declare that the research was conducted in the absence of any commercial or financial relationships that could be construed as a potential conflict of interest.

## Publisher’s Note

All claims expressed in this article are solely those of the authors and do not necessarily represent those of their affiliated organizations, or those of the publisher, the editors and the reviewers. Any product that may be evaluated in this article, or claim that may be made by its manufacturer, is not guaranteed or endorsed by the publisher.

## References

[1] Kilgore KP Lee MS Leavitt JA Mokri B Hodge DO Frank RD. Re-Evaluating the Incidence of Idiopathic Intracranial Hypertension in an Era of Increasing Obesity. Ophthalmology (2017) 124(5):697–700. doi: 10.1016/j.ophtha.2017.01.006 28187976 PMC5637544

[2] Mollan SP Aguiar M Evison F Frew E Sinclair AJ. The Expanding Burden of Idiopathic Intracranial Hypertension. Eye (2019) 33(3):478–85. doi: 10.1038/s41433-018-0238-5 PMC6460708 30356129

[3] Chen J Wall M. Epidemiology and Risk Factors for Idiopathic Intracranial Hypertension. Int Ophthalmol Clin (2014) 54(1):1–11. doi: 10.1097/IIO.0b013e3182aabf11 PMC3864361 24296367

[4] Mollan SP Mytton J Tsermoulas G Sinclair AJ. Idiopathic Intracranial Hypertension: Evaluation of Admissions and Emergency Readmissions Through the Hospital Episode Statistic Dataset Between 2002-2020. Life (2021) 11(5):417. doi: 10.3390/life11050417 34063037 PMC8148005

[5] Friesner D Rosenman R Lobb BM Tanne E. Idiopathic Intracranial Hypertension in the USA: The Role of Obesity in Establishing Prevalence and Healthcare Costs. In Obes Rev (2011) Vol. 12:e372–e380). doi: 10.1111/j.1467-789x.2010.00799.x 20804521

[6] Nanavati AJ Prabhakar S. Fast-Track Surgery: Toward Comprehensive Peri-Operative Care. Anesth Essays Res (2014) 8(2):127–33. doi: 10.4103/0259-1162.134474 PMC4173620 25886214

[7] Mollan SP Davies B Silver NC Shaw S Mallucci CL Wakerley BR. Idiopathic Intracranial Hypertension: Consensus Guidelines on Management. J Neurol Neurosurg Psychiatry (2018) 89(10):1088–100. doi: 10.1136/jnnp-2017-317440 PMC6166610 29903905

[8] Bouffard MA. Fulminant Idiopathic Intracranial Hypertension. Curr Neurol Neurosci Rep (2020) 20(4):8. doi: 10.1007/s11910-020-1026-8 32219578

[9] Gurney SP Ramalingam S Thomas A Sinclair AJ Mollan SP. Exploring The Current Management Idiopathic Intracranial Hypertension, And Understanding The Role Of Dural Venous Sinus Stenting. Eye Brain (2020) 12:1–13. doi: 10.2147/EB.S193027 32021528 PMC6969694

[10] Satti SR Leishangthem L Chaudry MI. Meta-Analysis of CSF Diversion Procedures and Dural Venous Sinus Stenting in the Setting of Medically Refractory Idiopathic Intracranial Hypertension. AJNR. Am J Neuroradiol (2015) 36(10):1899–904. doi: 10.3174/ajnr.A4377 PMC7965019 26251432

[11] Leishangthem L SirDeshpande P Dua D Satti SR. Dural Venous Sinus Stenting for Idiopathic Intracranial Hypertension: An Updated Review. J Neuroradiol J Neuroradiologie (2019) 46(2):148–54. doi: 10.1016/j.neurad.2018.09.001 30219337

[12] Radvany MG Solomon D Nijjar S Subramanian PS Miller NR Rigamonti D. Visual and Neurological Outcomes Following Endovascular Stenting for Pseudotumor Cerebri Associated With Transverse Sinus Stenosis. J Neuro-Ophthalmol (2013) 33(2):117–22. doi: 10.1097/WNO.0b013e31827f18eb 23502837

[13] Nicholson P Brinjikji W Radovanovic I Hilditch CA Tsang ACO Krings T. Venous Sinus Stenting for Idiopathic Intracranial Hypertension: A Systematic Review and Meta-Analysis. J Neurointerv Surg (2019) 11(4):380–5. doi: 10.1136/neurintsurg-2018-014172 30166333

[14] Ahmed RM Zmudzki F Parker GD Owler BK Halmagyi GM. Transverse Sinus Stenting for Pseudotumor Cerebri: A Cost Comparison With CSF Shunting. AJNR. Am J Neuroradiol (2014) 35(5):952–8. doi: 10.3174/ajnr.A3806 PMC7964533 24287092

[15] Fonseca PL Rigamonti D Miller NR Subramanian PS. Visual Outcomes of Surgical Intervention for Pseudotumour Cerebri: Optic Nerve Sheath Fenestration Versus Cerebrospinal Fluid Diversion. Br J Ophthalmol (2014) 98(10):1360–3. doi: 10.1136/bjophthalmol-2014-304953 24820047

[16] Murphy S Friesner DL Rosenman R Waslo CS Au J Tanne E. Emergency Department Utilization Among Individuals With Idiopathic Intracranial Hypertension. Int J Health Care Qual Assur (2019) 32(1):152–63. doi: 10.1108/IJHCQA-04-2017-0060 PMC6750901 30859875

[17] Liu A Elder BD Sankey EW Goodwin CR Jusué-Torres I Rigamonti D. Are Shunt Series and Shunt Patency Studies Useful in Patients With Shunted Idiopathic Intracranial Hypertension in the Emergency Department? Clin Neurol Neurosurg (2015) 138:89–93. doi: 10.1016/j.clineuro.2015.08.008 26302017

[18] Schwenk W Müller JM. [What Is “Fast-Track”-Surgery?]. Deutsche medizinische Wochenschrift (2005) 130(10):536–40. doi: 10.1055/s-2005-863090 15744648

[19] Kehlet H Wilmore DW. Evidence-Based Surgical Care and the Evolution of Fast-Track Surgery. Ann Surg (2008) 248(2):189–98. doi: 10.1097/SLA.0b013e31817f2c1a 18650627

[20] Krajcer Z Ramaiah VG Henao EA Nelson WK Moursi MM Rajasinghe HA. Comparison of Perioperative Costs With Fast-Track vs Standard Endovascular Aneurysm Repair. Vasc Health Risk Manage (2019) 15:385–93. doi: 10.2147/VHRM.S210593 PMC6731968 31564888

[21] Collins CI Hasan TF Mooney LH Talbot JL Fouraker AL Nelson KF. Subarachnoid Hemorrhage “Fast Track”: A Health Economics and Health Care Redesign Approach for Early Selected Hospital Discharge. Mayo Clinic Proc Inov Qual Outcomes (2020) 4(3):238–48. doi: 10.1016/j.mayocpiqo.2020.04.001 PMC7283927 32542215

[22] Lobatto DJ Vliet Vlieland TPM van den Hout WB de Vries F de Vries AF Schutte PJ. Feasibility, Safety, and Outcomes of a Stratified Fast-Track Care Trajectory in Pituitary Surgery. Endocrine (2020) 69(1):175–87. doi: 10.1007/s12020-020-02308-2 PMC7343751 32361869

[23] van Beekum C Stoffels B von Websky M Ritz J-P Stinner B Post S. [Implementation of a Fast Track Program: Challenges and Solution Approaches]. Der Chirurg; Z fur alle Gebiete der operativen Medizen (2020) 91(2):143–9. doi: 10.1007/s00104-019-1009-y 31372676

